# Envenomations by *Bothrops* and *Crotalus* Snakes Induce the Release of Mitochondrial Alarmins

**DOI:** 10.1371/journal.pntd.0001526

**Published:** 2012-02-21

**Authors:** Irene Zornetta, Paola Caccin, Julián Fernandez, Bruno Lomonte, José María Gutierrez, Cesare Montecucco

**Affiliations:** 1 Dipartimento di Scienze Biomediche, Università degli Studi di Padova, Padova, Italy; 2 Facultad de Microbiología, Instituto Clodomiro Picado, Universidad de Costa Rica, San José, Costa Rica; Liverpool School of Tropical Medicine, United Kingdom

## Abstract

Skeletal muscle necrosis is a common manifestation of viperid snakebite envenomations. Venoms from snakes of the genus *Bothrops*, such as that of *B. asper*, induce muscle tissue damage at the site of venom injection, provoking severe local pathology which often results in permanent sequelae. In contrast, the venom of the South American rattlesnake *Crotalus durissus terrificus*, induces a clinical picture of systemic myotoxicity, i.e., rhabdomyolysis, together with neurotoxicity. It is known that molecules released from damaged muscle might act as ‘danger’ signals. These are known as ‘alarmins’, and contribute to the inflammatory reaction by activating the innate immune system. Here we show that the venoms of *B. asper* and *C. d. terrificus* release the mitochondrial markers mtDNA (from the matrix) and cytochrome c (Cyt *c*) from the intermembrane space, from *ex vivo* mouse *tibialis anterior* muscles. Cyt c was released to a similar extent by the two venoms whereas *B. asper* venom induced the release of higher amounts of mtDNA, thus reflecting hitherto some differences in their pathological action on muscle mitochondria. At variance, injection of these venoms in mice resulted in a different time-course of mtDNA release, with *B. asper* venom inducing an early onset increment in plasma levels and *C. d. terrificus* venom provoking a delayed release. We suggest that the release of mitochondrial ‘alarmins’ might contribute to the local and systemic inflammatory events characteristic of snakebite envenomations.

## Author Summary

Every year, hundreds of thousands of people in tropical and sub-tropical areas of the world are bitten by poisonous snakes and may develop permanent damages. This is a major tropical disease which is largely neglected by scientific and clinical investigators. Snakes of *Bothrops* and *Crotalus* genus are responsible of most cases in Latin America. Here for the first time, we have shown that these venoms cause the release of both mitochondrial DNA and cytochrome *c*, two well known alarmins. Moreover, the kinetic of these processes are in agreement with the different pathophysiological profiles exhibited by *Bothrops* and *Crotalus* envenomations. These elements suggest a correlation between snake evenomations and sterile inflammatory syndrome. Alarmins are reported to have a fundamental role in innate immune response and inflammation; they might contribute to the local and systemic inflammatory events characteristic of these envenomations opening a new prospective in the study of these complex pathologies.

## Introduction

Snakebite envenomation is a neglected tropical disease that affects each year hundreds of thousands of individuals in tropical and sub-tropical areas of the world [1] [2]. In addition to death, many snake bitten patients develop permanent physical and psychological sequelae which greatly affect their quality of life [3] [4] [5] [6].

In the Americas, species of the family Viperidae are responsible for the vast majority of snakebite envenomations [7] [5] [8]. In Latin America, most cases are inflicted by species of the genus *Bothrops*, among which the lance-head vipers *B. asper* and *B. atrox* are very important in Central and South America, respectively [7]. In addition, the rattlesnake *Crotalus durissus* is notorious in South America for inflicting severe envenomations [7] [9]. The pathophysiology of envenomations by *B. asper* (BaV) and *C. durissus* (CdV) and their predominant toxins has been investigated at experimental and clinical levels [10] [11] [12] [13] [9]. These venoms induce strikingly different pathophysiological patterns. BaV, similarly to other *Bothrops* spp venoms, induce local pathological alterations associated with edema, myonecrosis, dermonecrosis, blistering and hemorrhage [12]. In addition, systemic alterations, i.e. coagulopathies, hemorrhage, acute renal failure and cardiovascular shock, may ensue in moderate and severe cases [11] [13]. Such a complex array of local and systemic alterations is mostly induced by the action of metalloproteinases, phospholipases A_2_ (PLA_2_) and PLA_2_ homologues, and serine proteinases, among other components [12] [13] [14] [15]. These envenomations present prominent local inflammatory response, associated with the activation of innate immune mechanisms, which might contribute to the pathogenesis of tissue damage [16].

In contrast to the effects of BaV, the pathophysiological manifestations induced by CdV are characterized by minor local alterations and prominent systemic effects, mostly neurotoxicity, systemic myotoxicity, i.e. rhabdomyolysis, acute renal failure and coagulopathies [9]. Around 60% of CdV is comprised by the dimeric PLA_2_ complex ‘crotoxin’ [17], which is composed by a basic PLA_2_ chain, crotoxin B, and a non-enzymatic acidic subunit, crotoxin A or crotapotin [18]. Cotapotin prevents the binding of crotoxin B subunit to non-specific sites and thus contributes to the high toxicity of this toxin [18]. Crotoxin exerts presynaptic neurotoxicity and systemic myotoxicity, which results in the release of large amounts of myoglobin from damaged muscle fibers, with the consequent impact on the kidney, provoking acute renal failure, which is a common finding in envenomations by this species [19]. Thus, envenomations by BaV and CdV represent different paradigms of tissue damage which greatly differ in the extent of the local inflammatory and pathological responses and in the systemic manifestations. On the basis of such different pathophysiological patterns, these venoms constitute valuable experimental tools to assess various aspects of local and systemic muscle damage and inflammation.

Snakebite envenomations trigger complex pathogenetic processes that include a range of defense reactions in the bitten organism, whose mechanisms are ill known, but resemble in several aspects muscle trauma [20] [21]. It has been long known that following tissue injury such as mechanical traumas,there is a massive release of molecules that act as “danger signals”, activating the host response [22] ATP is the prototype of these molecules, and when it is released from damaged or stressed cells to the extracellular space it acts via binding to an array of purinergic receptors [23] [24] [25] [26]. We have recently found that both Asp49 and Lys49 PLA_2_ myotoxins from BaV induce the release of ATP and K^+^ from muscles *ex vivo* and muscle cells in culture, and that this ATP extends the range of damage caused by these toxins [27]. ATP plays also a major role in the pathogenesis and symptoms following traumatic accidents [28] [29]. Very recently, it was demonstrated that traumatic injuries also induce the release of DNA and N-formylated proteins from the mitochondria of damaged tissues [30]. These molecules, known as ‘alarmins’ [31] [32] are able to activate neutrophils because they are recognized via receptors highly conserved during evolution as they are devoted to the innate immune response towards microbial molecules [30] [33]. On the basis of the pathological manifestations induced by BaV and CdV, we have investigated whether envenomations by these archetypal venoms induce the release of mitochondrial molecules, by evaluating the release of mitochondrial DNA and cytochrome *c* in isolated skeletal muscles and after *in vivo* injection of the venoms in mice.

## Materials and Methods

### Venoms and animals

The venom of *B. asper* was a pool obtained from more than 40 adult specimens collected in the Pacific region of Costa Rica; venom was lyophilized and stored at -20°C. Venoms were dissolved in 10 mM Hepes and 150 mM NaCl with 50% glycerol and sterilized by filtration through 0.22 µm GV Durapore® (Millipore). *C. d. terrificus* venom was from Latoxan (Valence, France). CD-1 mice received standard food and had free access to food and water.

### Ethics statement

All experimental procedures involving animals were carried out in accordance with the Italian Animal Welfare Act and were approved by the local authority veterinary service.

### Muscle Isolation and preparation of mtDNA

*Tibialis anterior* muscles were isolated from CD-1 mice weighing 25–30 g and immediately transferred to vials containing 1 ml of incubation buffer (139 mM NaCl, 12 mM NaHCO_3_, 4 mM KCl, 2 mM CaCl_2_, 1 mM MgCl_2_, 1 mM KH_2_PO_4_, and 11 mM glucose, pH 7.4) oxygenated (95% O_2_, 5% CO_2_) at 37°C. BaV and CdV (50 µg/ml) were added to the bath for the indicated time period, and the same volume of vehicle alone (10 mM Hepes and 150 mM NaCl with 50% glycerol) was added to the contralateral muscle used as control. At the end of incubation time, the supernatants were treated with RNAse A (100 mg/ml) to avoid RNA contamination and mtDNA was extracted using DNeasy Blood & Tissue kit (Qiagen) following manufacturer's instructions.

### Mice injection and plasma mtDNA preparation

Groups of three CD-1 mice were injected intramuscularly into the right leg with BaV (5 mg/kg), CdV (0.15 mg/kg) or the same volume of vehicle. The different dosages due to the higher toxicity of CdV were chosen to ensure that all animals survived during a 24 hr period. After 1 hr or 24 hrs, mice were sacrificed and immediately bled using up to 10 U/ml of heparin (Roche) to avoid interference with the following analyses. Plasma was separated and processed for mtDNA extraction using DNeasy Blood & Tissue kit (Qiagen) following manufacturer's instructions, after the treatment with RNAse A as previously described.

### Real-time qPCR

Primers for mouse cytochrome B (forward 5′-TGATGAAACTTTGGGTCCCTTC-3′ and reverse 5′-ATAAGCCTCGTCCGACATGAA-3′), and mouse cytochrome C oxidase subunit III (forward 5′-GTCCCACTACTTAATACTTC-3′ and reverse 5′-GGTGAAGTAAAGTCCTAGT-3′) were synthesized by Invitrogen. Primer sequences have no significant homology with DNA found in any bacterial species published on BLAST. Samples that produced no PCR products after 33 cycles were considered ‘undetectable’. Real-time qPCR was performed using iCycler® thermal cycler (Bio-Rad). Amplification conditions were: 10 min at 95°C, 40 cycles: 10 sec at 95°C, 30 sec at 52°C. A melting curve analysis, consisting of an initial step at 65°C for 10 sec and a slow elevation of temperature (0.5°C/s) to 95°C, was performed at the end of the amplification cycles to check for the absence of primer dimers and non-specific products using iQ SYBR Green supermix (Biorad). Results were expressed as detection folds of target genes in venom treated samples compared to control samples.

### Western blotting and cytochrome c (Cyt *c*) release detection

*Tibialis anterior* muscles were isolated from CD-1 mice and immediately transferred to vials containing 1 ml of the previously described oxygenated incubation buffer at 37°C. BaV or CdV venoms (50 µg/ml) were added to the bath for the indicated time period, and the same volume of vehicle alone was added to the contralateral muscle used as control. Samples of incubation medium were taken at different time points and protein concentrations were determined with the BCA Protein Assay (Pierce). The same quantification was done on plasma samples taken from injected mice. For each sample, 2.5 µg of total protein (for *ex vivo* experiments) or 50 µg (for plasma analysis) were loaded on 12% SDS-polyacrylamide gels, run at room temperature at 20 mA and transferred at 200 mA to a nitrocellulose in a refrigerated chamber. Membranes were incubated with an anti-cytochrome C antibody (BD Biosciences) following manufacturer's instructions. Chemiluminescence was developed with Luminata™ Crescendo (Millipore) or ECL Advance western blotting detection system (GE Healthcare), and emission was measured with ChemiDoc XRS (Bio-Rad). Band intensities were quantified on the original files with the software Quantity One (Bio-Rad). None of the bands reached signal saturation.

## Results

Envenomations by viperid snakes, such as those induced by *B. asper*, are often characterized by prominent tissue damage and inflammation at the site of venom injection. These venoms contain myotoxic PLA_2_s and PLA_2_ homologues which induce rapid alterations to the plasma membrane of the skeletal muscle cells, followed by irreversible cell injury [34] [35]. The venom of *C. d. terrificus* contains large amounts of the neuro- and myotoxic PLA_2_ complex crotoxin, which induces local and systemic myotoxicity [36] [37]. These myotoxins are not known to enter into cells, but they do cause rapid change in plasma membrane permeability, evidenced by a rapid loss of cytosolic markers, e.g. LDH and CK [38] [39] [40] [41]. The incubation of mouse *tibialis anterior* muscle with either BaV or CdV resulted in a similar extent of LDH release (Fig. S1).

**Figure 1:**
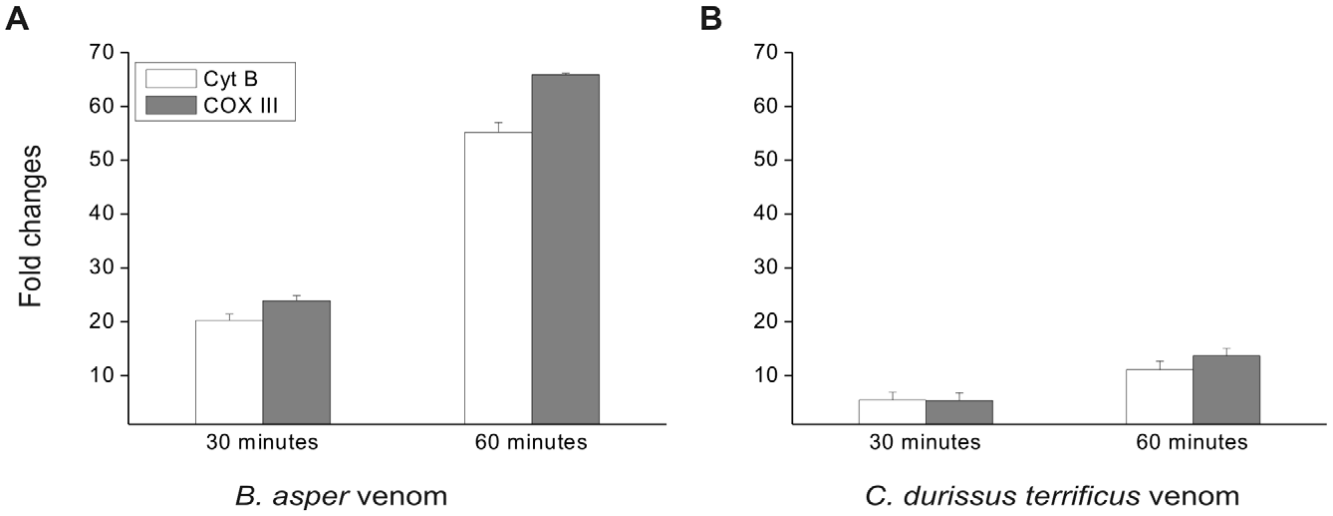
*B. asper* and *C. durissus terrificus* venoms induce the release of mtDNA. mtDNA released after *ex vivo* treatment (as described in Material and methods section) with snake venoms was determined by qPCR, using vehicle treated mice muscles as controls. Mean ± SD fold changes (treated samples/controls) in DNA coding for Cyt B and COX III relative to isolated *tibials anterior* mice muscles treated with (A) 50 µg/ml *B. asper* venom (BaV) or with (B) 50 µg/ml *C. durissus terrificus* venom (CdV) for 30′ and 60′ at 37°C in oxygenated physiological solution. Data represent the means of 6 independent experiments.

**Figure 2:**
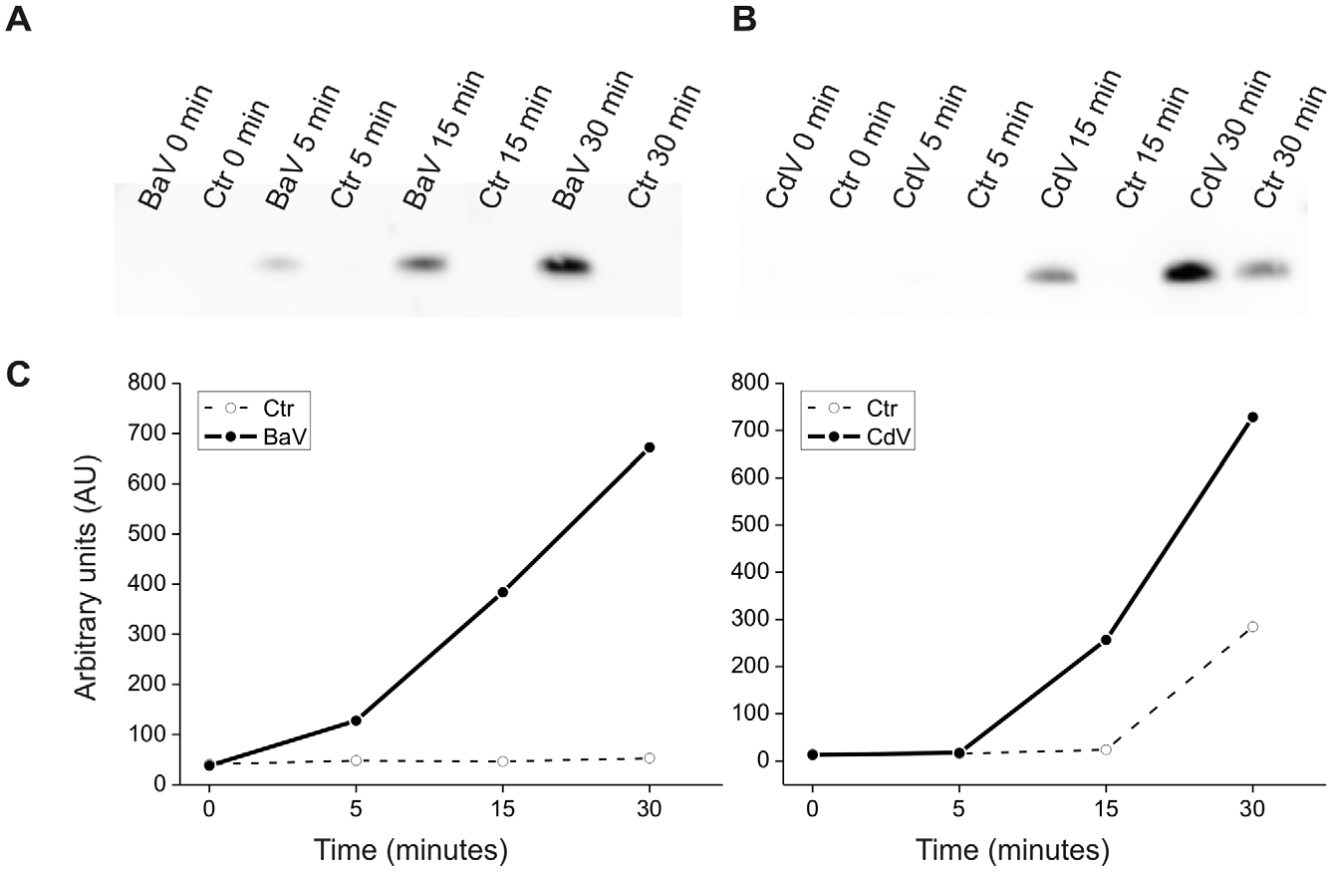
*B. asper* and *C. durissus terrificus* venoms induce Cytochrome *c* release. Time course of Cyt *c* release from isolated *tibialis anterior* mice muscles in the incubation medium after addition of BaV (A) or CdV (B). The protein concentrations were determined and 2.5 µg of total proteins were loaded in each lane. Western blots depicting the time course of Cyt *c* release (A) after BaV treatment (50 µg/ml) or (B) after CdV (50 ug/ml) and the same volume of vehicle as control. (C) The graphs report the quantitative analysis of the kinetics of Cyt *c* release induced by venoms (black lines) and controls (dotted lines). The intensity of each band was determined using the software Quantity One (Bio-Rad) The blots and their quantification show one representative experiment (n≥3).

Recently, it was shown that traumatic injuries induce the release of mitochondrial DNA, which, owing to its similarity to bacterial DNA, causes activation of innate immune cells [30]. This finding prompted us to test the possibility that BaV and CdV are able to induce the same effects. We used quantitative real-time PCR to evaluate mtDNA release from isolated *tibialis anterior* muscles treated with BaV or CdV. Fig. 1 shows that both venoms rapidly induce a rapid release of mtDNA from the treated muscle. BaV is more effective than CdV in both cases the amount of released mtDNA increased with time. Mitochondria are compartmentalized by two highly specialized membranes which create two separate spaces: the matrix, where mtDNA is located, and the intermembrane space, where Cyt *c* is present. Both mtDNA and Cyt *c* can act as alarmins [42] therefore we also investigated the release of Cyt *c*. Fig. 2 shows that, following treatment of *tibialis anterior* muscles with BaV or CdV, Cyt *c* is rapidly released; its presence in the medium is detectable soon after 15 min from addition of venoms to the bathing solution.

**Figure 3:**
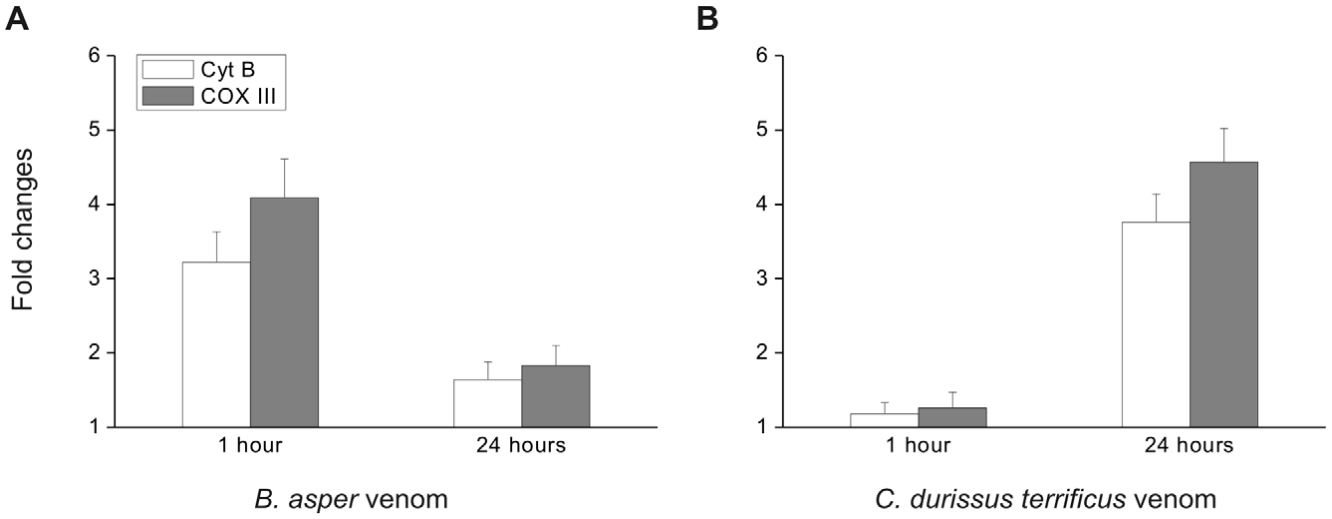
Envenomations by BaV and CdV result in blood circulation of mtDNA with different kinetics. Data obtained with qPCR show that mtDNA is released by intramuscular injection of the venoms. Each bar represents the fold changes of Cyt B and COX III mitochondrial genes in the plasma of mice treated (A) with BaV (5 mg/kg) or (B) with CdV (0.15 mg/kg). mtDNA was sampled from mice plasma 1 h and 24 h post-injection as indicated. Data represent the means of 3 independent experiments.

In order to extend the analysis of alarmin release in the context of the whole animal, venoms were injected intramuscularly in mice, followed by the quantification of mtDNA and Cyt *c* in the plasma. Mitochondrial alarmins were detected in the plasma of envenomated mice, as it has been described for traumatized patients [30]. The amount of mtDNA in the plasma was measured by real-time PCR after 1 and 24 hrs from injection. Fig. 3 shows that the pattern of mtDNA increase in the plasma differs among the two venoms, with a higher peak at 1 hr in the case of BaV injection and a higher concentration at 24 hrs for CdV.

**Figure 4:**
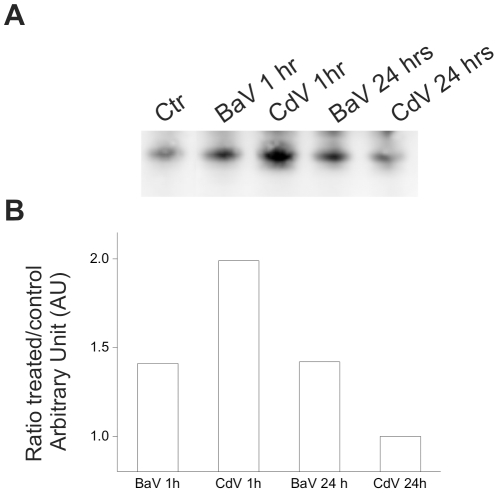
Plasma Cytochrome *c* release after envenomations by BaV and CdV. Time course of Cyt *c* release in the plasma of mice treated with BaV and CdV was performed as described in Materials and methods. The protein concentrations were determined in small samples and 50 µg of total proteins were loaded in each lane. (A) Western blots depicting the time course of Cyt *c* release after injection of either BaV (5 mg/kg) or CdV (0.15 mg/kg), or the same volume of vehicle as control. (B) The graphs report the relative quantification analysis of the kinetics of Cyt *c* release induced by venoms, as compared to the control. The intensity of each band was determined using the software Quantity One (Bio-Rad). The blot and its quantification show one representative experiment.

Cyt *c* release was also detected in the blood of patients who experience massive cell death, such as in systemic inflammatory response syndrome [43]. We next used Western blotting to detect Cyt *c* because other immunoassays, such as sandwich ELISA, may not give a reliable response in the presence of serum. Indeed, serum leucine-rich alpha-2-glycoprotein-1 binds to Cyt *c* and inhibits its recognition by specific antibodies [44], Such interference can be bypassed by using Western blotting. Fig. 4 shows that Cyt *c* was increased in the plasma of mice injected with either BaV or CdV 1 hr after injection, and its levels remained high after 24 hrs compared to control mice.

## Discussion

Muscle injury almost invariably leads to release of intracellular molecules, some of which constitute alarm signals which induce an innate immune reaction following their binding to specific receptors in various cell types [31]. This represents a general and fundamental defense response [45] [46]. The first of such intracellular molecules to be identified was ATP, which binds to a variety of purinergic receptors [25]. Very recently, mitochondria have emerged as a source of alarmins, such as mtDNA, as well as N-formylated proteins which bind to Toll-like receptors and to the formyl-peptide receptors and induce neutrophil activation [30]. These molecules are quite similar to their bacterial counterparts which are well characterized inducers of innate immune reactions [47] [48] [49]. Activation of neutrophils contributes to a variety of inflammatory and tissue repair events. Here, we have shown that BaV and CdV rapidly induce the release of both mtDNA and of Cyt *c* which can be detected both in the plasma of injected mice and in the medium of isolated muscles after incubation with the venoms. It has been previously reported an important cytokine release in envenomated mice, therefore we did not analyzed this aspect of immune response [50] [51] [16] [52] [53] [54] [55].

The two venoms were found to differ significantly in their kinetics of alarmin release in injected mice. BaV was found to be very rapid in inducing the release of both types of mitochondrial molecules, whilst CdV was rapid in causing Cyt c release, but slower in that of mtDNA. As mtDNA is located in the matrix and Cyt *c* in the inter-membrane space, these data highlight possible differences in the way these venoms affect mitochondria in muscle fibers. In *ex vivo* experiments using the *tibialis anterior* muscle, BaV induces a more drastic damage of mitochondria with alteration of permeability of both the outer and the inner membranes, whilst CdV seems to damage predominantly the outer membrane and, to a lesser extent and later on, the inner membrane. In the same model, both venoms induce a release of LDH from cytosol, which was more pronounced in the case of BaV.

The basis for the differences in mtDNA release by these venoms is puzzling, since both their main myotoxic components, i.e. *B. asper* PLA_2_ myotoxins and *C. d. terrificus* crotoxin act primarily by disrupting the integrity of skeletal muscle sarcolemma, inducing a calcium influx that generates a series of intracellular degenerative events [34]. Some of the most notorious ultrastructural consequences of the action of these toxins are observed in mitochondria, such as high amplitude swelling, disruption of cristae, appearance of flocculent densities and precipitates of hydroxyapatite [37] [56]. Despite these ultrastructural similarities in damaged mitochondria, our observations are likely to reveal more subtle differences in the mode and kinetics with which these venoms affect this organelle, a subject that needs to be further investigated. For instance, there might be variations in the release of mtDNA via inner-outer mitochondrial membrane specialized junction sites [57]. In addition, and perhaps most importantly, one should consider the involvment of other components of the two venoms in the envenomation process. For instance, viperid snake venoms, including those of *B. asper* and *C. durissus*, contain DNAses [58], which might degrade released mtDNA. Moreover, BaV myotoxins are able to affect types I, IIA and IIB muscle fibers, whereas crotoxin is more selective towards oxidative types I and IIA fibers [36]; since *tibialis anterior* muscle is predominantly constituted by type II fibers [59] such difference might have implications in the mtDNA release. Differences in the mechanism of action of crotoxin and BaV myotoxins were shown by their different myotoxic response to the pretreatment of animals with calcineurin [60], an observation that might be related to the variable specificity towards different muscle fiber types.

Our *in vivo* approch allowed the analysis of alarmin release in the whole animal, i.e. in a model that resembles the actual circumstances of snakebite. Intramuscular injection of these venoms in mice revealed marked differences in the kinetics of mitochondrial marker release. In the case of BaV, similar plasma concentration of Cyc *c* was observed at 1 and 24 hr, whereas the release of mtDNA was significantly higher at 1 hr. In contrast, CdV induce a higher Cyt c release at 1 hr, but a peak of mtDNA release at 24 hr. These differences can be interpreted in the light of previous observations on the myotoxic action of *Bothrops* sp myotoxins and crotoxin. The former induces predominantly local myotoxicity, i.e. muscle necrosis at the site of venom injection, with a very rapid increase in plasma CK activity, followed by a rapid drop. In contrast, crotoxin induces a more prolonged increment of CK activity in plasma, associated with systemic myotoxicity [36] [40]. The late increment in mtDNA in plasma is compatible with the predominantly systemic myotoxicity of CdV.

Our findings on the release of alarmins from muscle tissue damaged by these venoms have implications in terms of the local and systemic inflammatory events associated with snakebite envenomations. The rapid and higher release of mtDNA from muscles treated with *B. asper* venom correlates with the prominent local inflammatory scenario characteristic of tissue injected with this venom, in which there is increase of eicosanoids, cytokines, matrix metalloproteinases and other inflammatory mediators [16] [53] [54] [55] [50], and a prominent influx of neutrophils and macrophages [51] [52]. In this context, the role of mtDNA and other alarmins in eliciting such strong inflammatory response needs to be assessed. In contrast, in the case of CdV, local inflammatory events are minor, as shown at experimental and clinical levels, probably due to the anti-inflammatory activity of this venom [61] [62]. This may be also related with the observed delay in mtDNA release *in vivo* and with the lower release of this alarmin from muscle *ex vivo*. On the other hand, systemic manifestations of envenomations by *Bothrops* spp. are associated with evidence of systemic inflammatory events, as revealed by increments in the plasma levels of some cytokines and nitric oxide after the administration of a lethal dose of *B. asper* and *B. jararaca* venoms in mice [63] [50]. In the case of CdV, it is suggested that the drastic systemic myotoxicity induced by this venom, with the release of alarmins and other danger signals from damaged muscles, is likely to play a role in the onset of systemic inflammation, an issue that remains to be investigated. It is known that mitochondrial DAMPs are released following various types of tissue injury, causing systemic inflammation [42]. We hypothesize that, in addition to the direct action of snake venom components on various tissues, the release of mitochondrial alarmins from damaged cells is likely to contribute to the onset of local and systemic inflammatory events which, in severe envenomations, may induce manifestations that resemble those of systemic inflammatory response syndrome (SIRS) [64]. In the light of the emerging fundamental role of mitochondria in innate immune response, it would be important to characterize this interplay and the different alarmins that might be involved [65] [66]. This novel perspective of the action of snake venoms opens therapeutic windows of action aimed at reducing the effects of such alarmins as a way to decrease the severity of snakebite envenomations because it is possible that the injection of antibodies directed against mitochondrial DNA and cytochrome c given soon after envenomation may have therapeutic value.

## Supporting Information

Figure S1 **LDH release in** ***ex vivo*** **mice muscles.** *Tibialis anterior* muscles were uncovered by skin dissection, removed and placed in 1 ml of physiological solution containing 50 µg/ml of venom. LDH enzymatic activity was determined in the supernatants of *B. asper* (triangles) and *C. durissus terrificus* (squares) treated muscles for the indicated time points. Circles indicate the LDH activity in mock treated control muscles. Data represent the means of four independent experiments. The release of LDH is as an index of loss of membrane integrity.
(TIF)
