# Supplementary figures and images for: Envenomations by Bothrops and Crotalus Snakes Induce the Release of Mitochondrial Alarmins
Source: PLoS Negl Trop Dis. 2012 Feb 21;6(2):e1526. doi: 10.1371/journal.pntd.0001526 (PMC3283552; doi:10.1371/journal.pntd.0001526)

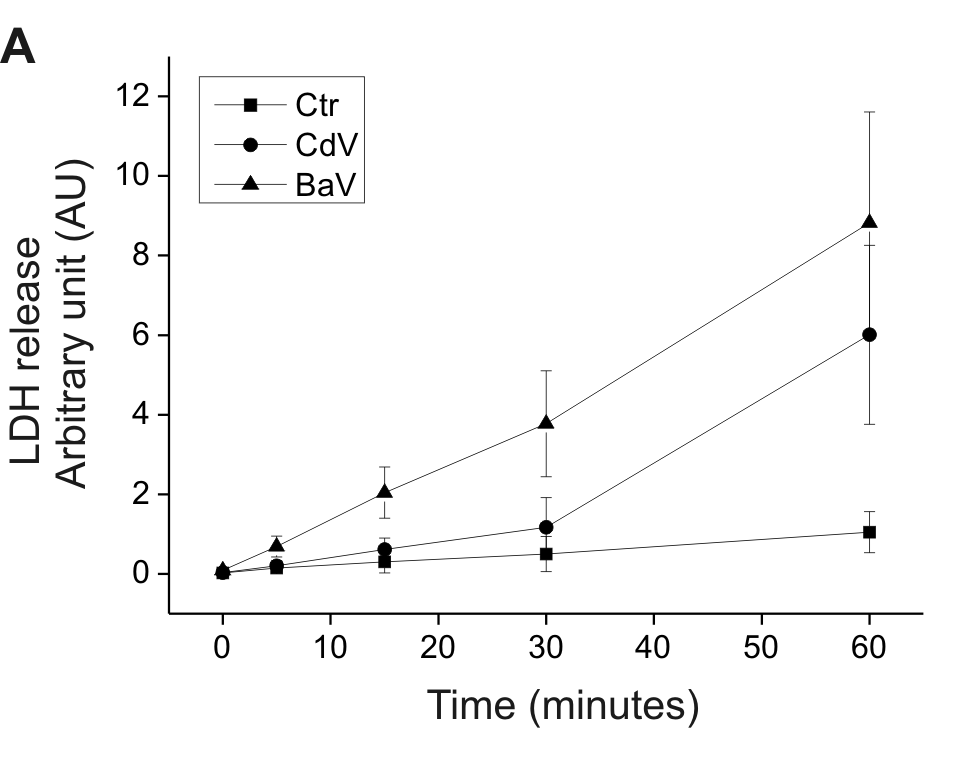

Supplement: Figure S1 — LDH release in ex vivo mice muscles. Tibialis anterior muscles were uncovered by skin dissection, removed and placed in 1 ml of physiological solution containing 50 µg/ml of venom. LDH enzymatic activity was determined in the supernatants of B. asper (triangles) and C. durissus terrificus (squares) treated muscles for the indicated time points. Circles indicate the LDH activity in mock treated control muscles. Data represent the means of four independent experiments. The release of LDH is as an index of loss of membrane integrity. (TIF) [file pntd.0001526.s001.tif]
